# Welfare without rent seeking? Buchanan’s demogrant proposal and the possibility of a constitutional welfare state

**DOI:** 10.1007/s10602-020-09321-7

**Published:** 2020-11-29

**Authors:** Otto Lehto, John Meadowcroft

**Affiliations:** Department of Political Economy, King’s College London, London, UK

**Keywords:** Buchanan, Demogrant, Universal basic income, Negative income tax, Welfare state, B29, B31, H53, I38

## Abstract

In a number of works, James M. Buchanan set out a proposal for a ‘demogrant’—a form of universal basic income that applied the principles of generality and non discrimination to the tax and the transfer sides of the scheme and was to be implemented as a constitutional rule outside the realm of day-to-day politics. The demogrant has received surprisingly little scholarly attention, but this article locates it in Buchanan’s broader constitutional political economy project and shows it was a logical application of his theoretical framework to the problem of inefficient and unfair welfare systems when reform to the basic institutions of majoritarian democracy was not forthcoming. The demogrant aims to end the problems of majority cycling and rent seeking that plague contemporary welfare states and therefore offers a model of welfare without rent seeking—a constitutional welfare state. We compare Buchanan’s demogrant model to other universal basic income and negative income tax models and consider the most important criticisms. We conclude that rescuing the demogrant model from relative obscurity would be a fruitful future task of applied constitutional political economy and public choice.

## Introduction

All contemporary democratic states make extensive transfer payments to qualifying citizens. On average social security payments—old age pensions, unemployment insurance, payments to families with children, and benefits to those unable to work through incapacity—equate to 20 per cent of the GDP of OECD countries.^1^ The modern welfare state no doubt reflects other-regarding concerns for those in need and ‘broadly shared citizen interests’ (Congleton 2007: 146), but its size and scope has long raised concerns that its principal beneficiaries are often the relatively wealthy (Cowen 2002; Goodin and Le Grand 1987; Bergh 2007), that it disincentivises personal investments in the social, cultural and human capital necessary for long-term exit from poverty and the realisation of a truly flourishing life (Cowen 2002; Evans 2017; Murray 1994), and that the employees of welfare state bureaucracies have become powerful vested interests who may use their significant discretion to prioritise their own ends over those of welfare recipients (Dunleavy 2017; Lipsky 2010; Zacka 2017). In the grammar of public choice, the welfare state has become a huge rent seeking enterprise in which a large proportion of the population uses the political process to secure benefits funded by others, while the resulting system traps many in genuine need into a lifetime of relative deprivation. In addition to the burden of these direct costs, the process of rent seeking consumes resources that could have been put to more productive use (Krueger 1974; Tullock 1967, 1971); resources are invested learning to navigate welfare systems, funding state bureaucracies able to process large numbers of claims, and protecting wealth from capture. Despite these significant costs, modern welfare states have not ameliorated concerns about deep injustices within democratic societies. On the contrary, a significant minority of citizens of affluent democratic societies are still born into relative poverty and disadvantage that they are more likely to reproduce than to escape (Bourguignon and Chakravarty 2019; Shelby 2016; Wolff and de-Shalit 2007). Conditional and means tested benefit schemes not only fail in the task of efficient poverty relief but are often bureaucratic, paternalistic, ‘punitive, meanspirited, … [and] callous’ in a way that may violate the dignity of poor people (Alston 2018). Accordingly, this would seem to be a salient moment to bring forward proposals for the reform of contemporary welfare states to protect the poor and needy from hardship, curtail rent seeking, and promote fairness.

Nobel laureate James M. Buchanan, a principal founder of public choice theory and constitutional political economy, set out a novel proposal for a ‘demogrant’ to address the inefficiencies and unfairness of the contemporary welfare state. The demogrant was a form of universal basic income with two unique characteristics: (1) the underlying principles of generality and non discrimination were to be applied to the tax side *and* the transfer side of the scheme; and (2) it was to be implemented as a constitutional rule outside the realm of day-to-day politics. The demogrant was intended to end the rent seeking generated by welfare states that provided targeted or means-tested benefits and liberate societies from the concomitant costs (Brennan and Buchanan 1985; Buchanan 1997, 2005; Buchanan and Congleton 1998).

The idea of a demogrant was discussed in several of Buchanan’s books and articles; it was incipient in his early works and reached maturity in his later contributions. Buchanan did not necessarily advocate the introduction of a demogrant—rather, in common with his general approach to the application of his ideas to real world policy-making, the demogrant was a proposal for others to consider as a possible solution to the problems of contemporary welfare states.^2^ However, next to the flat tax proposal and the balanced budget amendment, the demogrant model is one of the few policy proposals that Buchanan repeatedly came back to in his writings. The closest Buchanan came to explicitly advocating the demogrant was in one of his final intellectual contributions when he said that the nondiscrimination principle that justifies the demogrant, and therefore the demogrant itself, ‘may be widely understood and accepted as an appropriate normative guideline’ and that ‘it would surely be better to have such a nondiscrimination provision in the constitutional document itself than to ignore the continuing blatant violation of the generality norm in the workings of ordinary majoritarian politics’ (Buchanan 2005). The flat-tax-and-demogrant model reflected Buchanan’s (2005) commitment to the principles of generality and non discrimination and belongs to the normative ‘lessons of public choice theory’.

Despite the relative importance of the demogrant idea to Buchanan’s broader project it has been largely neglected in Buchanan scholarship, public choice theory and constitutional political economy. For example, Richard Wagner’s (2018) edited collection devoted to Buchanan’s work of fifty chapters spanning more than one thousand pages did not include a single reference to the demogrant. Niclas Berggren (2000) appears to have been the only scholar to hitherto attempt a serious evaluation of the proposal. This lack of attention would seem particularly remiss given the increasing political salience of basic income policies: Switzerland held a national referendum in 2016 on the introduction of an unconditional basic income in which 23 per cent of voters supported the idea; the US government’s unconditional stimulus payments of $1200 to every adult citizen and $500 for each dependent child in March 2020 as part of its response to the COVID-19 pandemic have some of the characteristics of a basic income. Basic income proposals have become part of the political discourse in many countries. Most notably, Finland ran a two-year UBI experiment in 2017–2018 on a randomly selected treatment group of 2000 unemployed people which resulted in small employment effects but significant increases in subjective wellbeing in comparison to the control group.^3^ Other randomized control trials (RCTs) on UBI and related programs have been conducted or are being planned in several countries around the world (Lehto 2018). The theoretical and empirical basis of UBI is subject to ongoing scrutiny. Buchanan’s demogrant proposal has the potential to make a unique contribution to these debates.

Our claim regarding the position of the demogrant in Buchanan’s oeuvre is that the demogrant plays a more significant role than is generally recognized in Buchanan scholarship. Nevertheless, its role may be relatively marginal compared to more fundamental constitutional questions and it was not necessarily advocated by Buchanan but rather examined as a theoretical model that could address the failures of welfare states, especially in relation to rent seeking and interest group politics. The demogrant proposal flows logically and repeatedly out of Buchanan’s early-to-mid-career work as an application of the constitutional principles of generality and nondiscrimination into contemporary welfare state governance. We contend that the demogrant proposal should be elevated in Buchanan scholarship from its current position of obscurity to one of greater prominence.

This article provides a systematic account of Buchanan’s demogrant model set out in the context of his wider intellectual project, relates the proposal to other basic income models, and then critically evaluates the idea. After this introduction, the article will locate the demogrant idea in Buchanan’s theory of politics as exchange and his long-standing concerns about the endurance of inherently exploitative political arrangements—the idea that societies can become trapped on the ‘off-diagonals’ of game theoretic models of social coordination and conflict. It will then set out the development of the demogrant idea through Buchanan’s career and show how it arose as a practical application of his commitment to the principles of generality and non discrimination. The article will then consider the relationship of the demogrant to other proposals for a universal basic income, notably those set out by F. A. Hayek, Milton Friedman and Charles Murray, before undertaking a critical evaluation. It will be concluded that there are reasons to be sceptical that a demogrant would be the panacea that Buchanan suggested given the difficulty of its implementation and the likely transaction costs involved, but the proposal nevertheless merits further attention from scholars working in public choice and constitutional political economy and those developing proposals for a universal basic income.

## Trapped on the off-diagonals: Buchanan’s model of politics as exchange and rent seeking

At the heart of Buchanan’s intellectual contributions was the contention that social order could be understood to emerge spontaneously from a process of exchange among individuals. An advanced economy emerged from the actions of individuals exchanging goods or services and similarly political institutions emerged from a process wherein individuals agreed to respect one another’s rights. These agreements were ‘exchanges’ in the sense that the agreement to respect rights was reciprocal. Multiple two-person agreements that came to encompass the whole of society constituted a social contract—the society-wide agreement of rights (Buchanan 1975, 1986, 1988; Gwartney and Holcombe 2014; Holcombe 2020; Marciano 2009; Vanberg 2018).

Buchanan (1975: 84) modelled ‘politics as exchange’ as a two-person Prisoner’s Dilemma shown in Table 1 in which two unequal individuals, A and B, choose to respect or not respect the rights of the other leading to four possible outcomes.

**Table 1:** Politics as exchange as a Prisoner’s Dilemma. *Source*: Buchanan (1975: 84)

	B Respects Rights	B Respects No Rights
A Respects Rights	Cell I 19, 7	Cell II 3, 11
A Respects No Rights	Cell III 22, 1	Cell IV 9, 2

Buchanan (1975) theorised that Cells II and III, the off-diagonal outcomes, were inherently unstable because the individual who received the worse pay-off in these cells was better off in Cell IV, so entry into Cell II or III automatically led to a move into Cell IV. Consequently, Buchanan argued that the actual choice A and B faced was along the diagonal from Cell I to Cell IV—between a social order of mutual respect and a Hobbesian war of all against all.

In Buchanan’s (1993, 1998; Buchanan and Lomasky 1984) later work, however, he became increasingly concerned with the idea that the off-diagonal outcomes—Cells II and III in Table 1—could be relatively stable. Politics may not necessarily produce either a social order of mutual respect where equal rights were respected or Hobbesian anarchy without rights. Rather, political outcomes characterised by asymmetrical rights could be sustained over a long period. For example, different individuals in the same polity could face markedly different tax burdens or receive very different entitlements from the state. Buchanan and Lomasky (1984) argued that these off-diagonal outcomes could be relatively stable because the prospect of Hobbesian anarchy was genuinely terrifying to some individuals who therefore accepted long-term, low-level exploitation within a stable legal-political order as a superior alternative to a return to the state of nature. This conclusion is surely not surprising and suggests that in Table 1 Buchanan over-estimated the pay-off for the weaker party in Cell IV. In Table 2 below the pay-off for B in Cell IV has been reduced to zero to reflect this individual’s fear of entering the state of nature, making Cell III a stable outcome.

**Table 2:** Politics as exchange as a Prisoner’s Dilemma with alternation to B’s pay-off in Cell IV

	B Respects Rights	B Respects No Rights
A Respects Rights	Cell I 19, 7	Cell II 3, 11
A Respects No Rights	Cell III 22, 1	Cell IV 9, 0

Buchanan and Congleton (1998: Chapter 3) developed this straightforward proposition into a more sophisticated analysis of the dynamics of majority cycling in a democracy that produced stable off-diagonal outcomes because each individual was a member of the winning coalition at some point and therefore was content for the process to continue. Indeed, this analysis probably captured an essential characteristic of democracies in which around half of national income was spent by government leading to significant churn of contribution to and receipt from government expenditure so that almost every citizen contributed along some margins and received along others and therefore it was impossible for any individual to know with certainty their precise net position. Hence, the ‘veil of uncertainty’ that Buchanan and Tullock (1962) identified in constitutional decision-making was also an important feature of ongoing post-constitutional politics.

It seems clear that the citizens of contemporary democracies prefer the off-diagonal outcomes produced by majoritarian decision-making to the diagonal cell in which no rights are respected. Even the weaker parties will be confident that they will receive some benefits from the political process, even if there are aware that others are more consistently members of winning coalitions and consequently gain more overall. But, importantly, the off-diagonal positions will nevertheless produce an overall inferior outcome compared to the diagonal in which all citizens have equal rights. In this diagonal, the rent seeking process and its concomitant opportunity costs have ceased leading to an overall superior outcome. Therefore, there are significant gains for society as a whole if it is possible to move from the off-diagonals to the superior diagonal—if rent seeking can be eliminated.

Buchanan (1997) and Buchanan and Congleton (1998) argued that the challenge of sticky off-diagonal outcomes could be overcome by the introduction of constitutional rules that removed the off-diagonal options. This would involve the application of the generality principle: the principle that all legislation was generally applicable to all citizens and therefore did not discriminate against any category of persons (Mueller 2018). The demogrant proposal was an important component of this attempt to apply the generality principle and enable contemporary democracies to escape the off-diagonals.

## Buchanan’s demogrant model

A ‘demogrant’ is a rather old-fashioned term for an unconditional income transfer paid to an entire demographic group in a nondiscriminatory fashion. The prefix is an abbreviation of ‘demographic’. The concept has two distinct applications: group-targeted and universal. In the group-targeted application, it refers to ‘a common grant available to all members of a demographic group without regard to any other condition or criterion’ (Okner 1973: 1). For example, a uniform old age pension is a type of group-targeted demogrant; it differs from conditional versions of old age pensions that further discriminate *within* the age group, for example by means-testing. In the most universal application, the demogrant refers to a uniform grant unconditionally given to all eligible members of the polity. Eligibility to the scheme is limited only by citizenship or permanent residence status, with no further criteria or conditions attached. The universal demogrant in this broadest sense is equivalent to what is today called a Universal Basic Income (UBI) or a Basic Income Guarantee (BIG). It also resembles Milton Friedman’s (1962) proposal for a Negative Income Tax (NIT) that guaranteed individuals a basic income from the state that would diminish as their earnings increased. Indeed, a UBI scheme married to a flat tax that effectively claws back the basic income from high income earners is identical to an NIT (Mankiw 2016).

The demogrant was popularized by the US Democratic Party presidential candidate George McGovern in his unsuccessful 1972 campaign. McGovern famously pledged an annual ‘demogrant’ of $1000 to every American citizen (roughly $6000 in 2020 dollars). McGovern’s proposal was inspired by a policy proposal developed by his economic advisor, James Tobin (1966). At the same time, the incumbent Republican presidential candidate Richard Nixon advocated a universal welfare program, the Family Assistance Plan (FAP), that loosely resembled the Negative Income Tax advocated by *his* economic advisor, Milton Friedman, who had popularized the NIT in his book *Capitalism and Freedom* (Friedman 1962). After Nixon’s victory, the Earned Income Tax Credit (EITC) program, which still exists, was born out of Nixon’s failure to pass his Negative Income Tax bill through the Senate (Moffitt 2003; Hungerford and Thiess 2013). The EITC program carries a family resemblance to the NIT model, but it eschews the latter’s universality and unconditionality. The NIT/demogrant debate of this time also spawned a string of UBI experiments in the US and Canada between 1968 and 1980 (Widerquist 2005).

Buchanan’s demogrant idea emerged at a time when a number of basic income schemes were on the agenda in American politics, but it should be said that Buchanan’s proposal was somewhat detached from these broader public and scholarly debates—he did not reference the existing literature on basic income proposals while creatively reframing the demogrant in terms of his own constitutional political economy paradigm. But Buchanan’s interest in the demogrant may nevertheless be understood as an indirect engagement with the work of Friedman and Tobin—two of the leading economists among Buchanan’s contemporaries. Buchanan must have also been aware of Hayek’s (1944, 1960, 1982) consistent support for a guaranteed minimum income, though, again, Buchanan did not explicitly discuss Hayek’s proposal. The fact that the demogrant idea was popularized by McGovern in 1972 may also be significant because we know that the early-1970s was a time of intense creativity for Buchanan that led to the formulation of the ideas that would dominate the remainder of his career (Buchanan 1992; Meadowcroft 2011: 29-30). Indeed, in *The Limits of Liberty* Buchanan (1975: 116) referenced McGovern’s 1972 campaign in the context of his analysis of citizen discontent with the status quo of American government and politics that would be an important theme of his work thereafter.

Buchanan’s discussion of the demogrant originated from the sustained development of the notions of generality and democratic consensus in his earlier work, notably *The Calculus of Consent*. Here, Buchanan and Tullock (1962: 166–171) argued that where a collective good was funded through an unequally distributed tax burden, or a general tax was used to fund an unequally distributed benefit, then the political process took on the characteristics of a strategic game in which individuals competed to assign benefits to themselves and/or impose costs on others. The solution to this dilemma, Buchanan and Tullock (1962: 170) contended, was a General Benefit-General Taxation Model in which all benefits and burdens applied equally to all, so that, ‘the collective-choice process does not take on the attributes of a game,’ and therefore, ‘the political process offers to the individual participant no opportunity to gain differential advantage at the expense of fellow participants’. In this context of this model, Buchanan and Tullock explained:

When the individual makes a decision… he must try to compare the advantages that he will secure from the availability of the collective good and the costs that he will undergo from the increase in the general tax. His behavior can exert no external effect, either in costs or benefits, on third parties.

In other words, in a simple formal model, generally funding and generally supplying collective goods removed the possibility of rent seeking. This analysis anticipated Buchanan’s long-standing interest in situations where ‘collective action takes on such characteristics of generality (that is, nondiscrimination)’ (Buchanan and Tullock 1962: 171). As we shall describe, the ‘double application’ of the generality principle to the tax and the benefit sides of the fiscal budget ultimately led to the unified demogrant and flat tax model explicitly developed in Buchanan’s later works. Buchanan and Tullock (1962: 171) lamented the fact that while many scholars ‘stress the importance of generality in the distribution of the tax burden among members of the social group… [n]o such principles have guided the distribution of public expenditure among the several possible uses’. The demogrant model arose out of the efforts of Buchanan and his co-authors to treat public expenditure and taxation symmetrically.

Buchanan’s engagement with the symmetric application of the principles of generality and non discrimination was further developed in the context of the idea of a fiscal constitution set out in *The Power to Tax* (1980), the first of two books co-authored with Geoffrey Brennan. Although this book did not explicitly discuss the demogrant, it pointed to the direction of the analysis of the transfer side of the budget that would eventually lead to Brennan and Buchanan’s articulation of the demogrant idea in *The Reason of Rules* (1985) five years later.

*The Power to Tax* was an analysis of the possible implementation of constitutional rules to prevent the state’s power to tax being used for exploitative purposes by electoral majorities (and also by the personnel of public sector bureaucracies). Brennan and Buchanan (1980: 12) argued a generality-uniformity constraint on the revenue side of the government budget that required individuals in similar circumstances to pay identical tax would make it more difficult to build a majority coalition because such a coalition required the inclusion of individuals with same the income and/or wealth as the outside minority liable to exploitation. Although a coalition willing to exploit a small minority of very wealthy people could still be constructed, this was unlikely to generate sufficient revenues to fund a modern state given that very high rates of taxation on a small group would quickly destroy their wealth or encourage its flight. Brennan and Buchanan’s (1980: 58) generality-uniformity constraint did not rule out progressive taxation—on the contrary they argued that a progressive tax structure (for example a tax rate of zero on initial earnings) embodied limits on the tax liabilities of citizens that could act as a significant constrain on the power to tax.

This analysis informed Brennan and Buchanan’s second major collaboration, *The Reason of Rules*, in which they first set out the demogrant proposal as a constitutional constraint on majoritarian democracy. The proposal was defined in the following terms:

[W]e shall suppose that the constitution restricts the taxing authorities to a single-rate income tax, no other restrictions being applied. Then we shall add the requirement that the income tax be levied at a uniform proportional rate on all individuals. We shall add the further restriction that transfers be paid in equal per capita amounts, in the form of a ‘demogrant’ (Brennan and Buchanan 1985: 135).

The two key features of the demogrant proposal can thus be identified. First, *symmetry between the tax and the transfer sides* of the government budget. Taxation and spending were treated equally and regarded as equally important co-determinants of the logic of the constitutional welfare state. Hence, the same operational principles of generality and non discrimination were applied symmetrically to the tax side and the expenditure side of the system. Second, the resulting fiscal constitution was a procedural *constraint* on the operation of the redistributive welfare state. The demogrant was designed to place the welfare system into the constitutional realm, away from day-to-day politics.

The idea that the demogrant can act as an operational constraint reflects Brennan and Buchanan’s (1985) broader focus on the abstract ‘rules of the game’ that are procedural determinants of post-constitutional politics. Rules constrain the capacity of the government, and ultimately of citizens acting through the government, to act in particular ways. The generality principle that underlies the demogrant enforces the regime of constitutional constraints that limits the leeway of the government to target grants on particular people or groups and target taxes at particular groups or people to pay for those grants. At the same time, on the constitutional level of hypothetical social contractual negotiation, the generality principle reflects universality as a democratic norm appropriate to a society of free and equal individuals.

Concretely, the demogrant entailed the benefit-side restriction that transfers be paid in equal per capita amounts. Whereas Brennan and Buchanan’s earlier work had not ruled out progressive taxation, the demogrant proposal involved a parallel tax-side restriction that income tax be levied at a uniform proportional rate on all individuals. On both the tax side and the benefit side, the demogrant demanded that the impact of rules was to be general and universal across the population. The function of this double restriction on the tax-and-transfer scheme was to affect the incentive structure of political agents (conceived as actively engaged in furthering their own interests through the political process) and remove the incentives that drove the majority cycle; where benefits and burdens were general and uniform it ceased to matter who was a member of the winning majority or the losing minority (Brennan and Buchanan 1985: 138–141).

The demogrant idea occupied a central place in a number of Buchanan’s later writings in which he accepted that majoritarianism was likely to remain the norm in liberal democratic states for the foreseeable future. In these works Buchanan focused on mechanisms to limit the negative consequences of majoritarian decision-making and ensure democracy promoted the welfare of *all* and not just the welfare of those individuals and groups able to build winning coalitions, rather than expose the pathologies of simple majority rule and present proposals for alternative decision-making rules, as he had done in earlier works like *The Calculus of Consent*. Buchanan’s concern, in the language of politics as exchange, became the achievement of fairness in a society trapped on the off-diagonals.

Buchanan’s proposal for a democracy that promoted the general welfare, rather than sectional interests, was an account of politics founded on principles, not interests. Buchanan and Congleton (1998: xii) argued that ‘politics requires an anchor in principle, lest it remain subject to the capricious forces of rotating coalition interests’ and proposed non discriminatory democracy under the generality norm as that foundational principle. This implied a distinction between procedural and substantive constraints on government action:

The critical distinction is procedural rather than substantive. Politics by principle constrains agents and agencies of governance to act nondiscriminatorily, to treat all persons and groups of persons alike, and to refrain from behaviour that is, in its nature, selective. Within the limits of such constraints, politics may do much or little (Buchanan and Congleton 1998: xii).

The procedural requirement of non discrimination did not forbid a high level of taxation nor a high level of redistributive government spending. It was therefore compatible with a welfare state that redistributed from rich to poor (given that contributions were proportionate to income but receipts were uniform), though of course incompatible with a welfare state that benefited particular occupational or sectional interests. Whether it more resembled a night watchman state or a welfare state in its substantive policies, the constitutional fiscal state was procedurally (but not substantively) constrained to follow a logic of operation derived from the ideal of generality and non discrimination (Vanberg 2011).

Buchanan and Congleton (1998: 34) reiterated that the application of the generality principle to both the tax side and the transfer side was intended to remove incentives for rent seeking:

[W]hen the [collective] choice set is constrained to incorporate symmetry or generality in the assignment of action to the separate parties… no participant has an incentive to invest resources in efforts to secure differential or discriminatory advantage at the expense of others in the collective enterprise.

The general, flat-tax, equal-demogrant scheme was anticipated to eliminate incentives for investment in efforts to qualify for transfers under separate and differential fiscal treatment for particular groups that might use public interest arguments to claim special entitlements. Of course, rent seeking could only be eliminated under the idealized constitutional conditions wherein the demogrant was a wholesale replacement of the welfare state and combined with strict constitutional limits on all supplementary and discretionary transfer schemes. This would be achieved by bundling together the various pre-existing redistributive policies into the single demogrant scheme and replacing in-kind transfers with cash transfers. As Buchanan and Congleton (1998: 149) wrote: ‘the budgetary mix under the political structure postulated here would be heavily weighted towards transfers and away from the financing of commonly valued public goods and services’.

The demogrant proposal would therefore end bureaucratic conditionality and means testing, even if this resulted in an increase in the overall government budget as payments would be made to individuals and families who would not qualify under a means-tested scheme: ‘The introduction of means testing will increase rent seeking or political inefficiency as it promises to reduce, somewhat, conventional excess burdens. Classical liberals, in particular, should beware of following a false god’ (Buchanan and Congleton 1998: 151).

It was not denied that means-testing had theoretical cost saving properties, but it was argued that these must be carefully balanced against the tendency of means-testing to increase rent seeking. Hence, the logic of the demogrant proposal was that putting an end to the use of majoritarian democracy as a rent seeking competition among interest groups would remove an important source of expansionary pressure on the government budget. While the demogrant proposal preserved majoritarianism, ‘it is not allowed to degenerate into the cross-group redistributive transfer absurdity that describes the “churning state”’ (Buchanan 1997: 172).

Buchanan (1997, 2005) acknowledged that the substantive realm within which the demogrant operated could legitimately be extended to include qualification criteria that were *sufficiently* general or met a public interest test based on need (not financial means). For example, tax-financed spending on pensions or medical care for the elderly might be adjudged to be non discriminatory since all citizens became equally eligible given that every person ages and age is not subject to behavioural manipulation. Indeed, ‘universal pensions’ are sometimes called ‘demogrants’ in the literature (OECD 2007: 44). But more difficult issues arose where benefits were differentially targeted on public interest grounds, but those benefits could not, by their nature, be classified as general, such as aid to the blind, deaf, or disabled. In these cases, the qualification criteria may be subject to some behavioural manipulation, concerning the qualifying degree of sight loss, hearing loss or disability. Buchanan (2005: unpaged) argued that, ‘No hard and fast line can be drawn here, and the apparent violation of any generality standard must be weighed against a meaningful interpretation of how much general interest is involved’. Hence, the generality principle would seem to be an instance of what Buchanan (1989) termed the ‘relatively absolute absolutes’—principles that should be regarded as absolute except in the very few cases where there was an overwhelming reason to disregard them.

Buchanan argued that if assistance to those in need could be elevated to the constitutional realm and thereby removed from the agenda of day-to-day majoritarian politics then democracy could promote the general welfare rather than group interests: ‘Legislative majorities would be empowered to set […] the size of the demogrant, but specific actions aimed at discriminating favourably or unfavourably […] would be out of bounds’ (Buchanan 1997: 171–172). Constitutionalization therefore guaranteed the (quasi-) permanence of the non discriminatory demogrant system. As Buchanan and Congleton (1998: 126) wrote:

[O]nce chosen and in place, the system will remain quasi-permanent and hence immune from period-to-period manipulation due to shifting majority coalitions. In this sense, constitutionalization implies that the transfer system, once chosen, is appropriately treated as ‘off the table’ for the interplay of ordinary majoritarian politics.

Buchanan further contended that the introduction of constitutional rules to limit the power of electoral majorities and end the concomitant rent seeking might legitimize redistributive democracy and increase public trust in its institutions: ‘The expressed public dissatisfaction with the modern welfare state may be traced, in part, to the failure to keep transfer programs within the limits of generality that […] promote the general welfare’ (Buchanan 1997: 179). Similarly, Buchanan and Congleton (1998: xii) argued that the introduction of the generality norm at the constitutional level could lead to the development of a politics that was widely perceived to operate for the betterment of all citizens, not just those able to capture the political process: ‘[P]olitics may be made “better” in the evaluation of all participants, if political action can be constrained constitutionally so as to meet more closely the generality norm’.

## Comparable proposals for generality in welfare and in taxation

There are many proposals for UBI, BIG and NIT and for ‘flatter’ tax systems that would require taxpayers to pay the same proportion of their income in tax. But Buchanan’s demogrant idea was unique in two ways: (1) in simultaneously applying the generality principle to both the tax and transfer sides of the welfare state in one unified proposal and (2) in emphasizing the constitutionalized operation of the tax-and-transfer system.

On the transfer side, it had strong similarities with the proposal for a guaranteed minimum income that Hayek advocated from *The Road to Serfdom* onwards (Hayek 1944, 1960, 1982). Hayek’s basic income proposal was intended to protect the poor and unfortunate against destitution while avoiding the problems of rent seeking and discrimination that Buchanan also identified. Hayek supported some mild form of conditionality, but libertarians have largely taken his model to be consistent with a UBI (Zwolinski 2015, 2019). He remained agnostic on whether basic income was best conceived as ‘an insurance against extreme misfortune’ or ‘a clear moral duty of all to assist’ the needy (Hayek 1982: 249). Hayek’s proposal, like Buchanan’s, was an explicit application of the generality principle to the provision of welfare; however, Hayek’s minimum income proposal was not explicitly linked to any particular tax system. And since Hayek did not specify how such a ‘certain minimum income for everyone, or a sort of floor below which nobody need fall even when he is unable to provide for himself’ (Hayek 1982: 395) should be implemented, other scholars must work out the details of how to implement a Hayekian UBI. The demogrant model suggests itself as a plausible candidate.

Similarly, Milton Friedman’s (1962) NIT proposal, like Buchanan’s demogrant, tried to combine the welfare and the tax sides of the equation into a single tax-and-transfer system where transfers are rebranded ‘negative taxes’ and taxes can be thought of as ‘negative transfers’. One distinction lies in the fact that the NIT is *directly* means tested and progressive whereas the demogrant scheme is neither. However, this is a distinction without substantive difference. Net transfers (benefits minus taxes) in the flat tax and demogrant system are *indirectly* means tested and progressive to the extent that high income people are made to pay back their demogrant in the form of proportional taxation. So, the overall *net* progressivity in both models is mathematically identical (Mankiw 2016). Likewise, both models embody the generality or nondiscrimination norm to some degree, but the similarity breaks down as soon as one looks at how stringently this norm is implemented. This marks the biggest difference between them: for while Friedman wished to use the NIT to *bundle together* a whole host of existing benefits and taxes, he did not explicitly argue for a constitutional *prohibition of* additional benefits or taxes like Buchanan.

Charles Murray’s (2016) UBI proposal also had similarities with Buchanan’s demogrant proposal, although he also did not cite Buchanan. In fact, Murray barely referred to the existing UBI literature, aside from Friedman. Murray explicitly proposed ‘a constitutional [UBI] amendment’ with striking resemblance to Buchanan’s non discriminatory constitutional amendment. Murray’s proposal is worth quoting at length since it exemplifies the sort of contemporary amendment of the welfare state along demogrant lines that Buchanan probably would have felt comfortable endorsing:

Henceforth, federal, state, and local governments shall make no law nor establish any program that transfers general tax revenues to some citizens and not to others, whether those transfers consist of money or in-kind benefits. All programs currently providing such benefits are to be terminated. The funds formerly allocated to them are to be used instead to provide every citizen with a Universal Basic Income beginning at age twenty-one and continuing until death (Murray 2016: 7).

On the benefit side, then, Murray’s basic income proposal may be the one that comes closest to Buchanan’s demogrant model. It seems plausible that this reflects Murray’s (1994, 2016) long-standing concern with the inefficiencies and unfairness of the American welfare system.

Despite its enduring place in Buchanan’s oeuvre, very few subsequent scholars in constitutional political economy or public choice have directly taken up the demogrant idea. Harmut Kliemt’s (1993, 1995) discussion of the possibility of a constitutional universal basic income in the 1990s was one exception. Interestingly, while Kliemt’s analysis is very much in the spirit and tradition of Virginian political economy, he did not directly cite Buchanan’s model. Kliemt (1993: 167–169) entertained the creation of a system of ‘social dividend’ as the constitutional foundation of a ‘minimum welfare state’. Such a state would be consistent with the rule of law and equality of treatment as much as a minimal (non-welfare) state. Kliemt (1995) also discussed the close relationship between the rule of law and the normative principle of ‘schematic equality’, which was essentially the same as the generality principle articulated by both Buchanan and Hayek. Kliemt (1995: 125) held that significant redistribution could be consistent with the principle of schematic equality, ‘as long as every individual would get exactly the same amount of money in a “citizens’ basic income scheme”, a negative income or a voucher system state sponsored redistribution would be in line with the principle’.

On the tax side, Buchanan’s contributions to public finance were an important influence on the intellectual milieu from which the modern flat tax movement developed. From the 1980s onward a number of established democracies took steps to ‘flatten’ their tax codes, removing marginal rates of taxation, and a number of post-socialist democracies introduced flat tax regimes. It can be difficult to isolate the impact of flat taxes given that these reforms were often introduced contemporaneously with other economic policies designed to liberalise the economy, but the evidence suggests that flat taxes have had a positive impact on tax compliance and economic growth, but have not led to an overall reduction in government spending (Hall and Rabuska 1995; Gorodnichenko et al. 2008; Keen et al. 2008).

Charles Delmotte (2020) has recently taken up the idea of non discriminatory taxation in debates concerning tax justice. Delmotte (2020: 63) has applied Buchanan’s generality principle to argue that ‘in order to liberate taxation from fiscal exploitation and to reconcile taxation and public finance with the general interest, taxation should follow the precepts of generality, of which tax uniformity is the best account’. But the logic of Buchanan’s argument dictates that the same principle of generality is also applied to the benefit side. Unfortunately, this idea has not been taken up by many public choice scholars. The corollary conclusion is to argue against welfare exemptions with the same rigour as one argues against tax exemptions. After all, both tax and welfare exemptions undermine generality and involve unwarranted discrimination that threatens to undermine the legitimacy of the constitutional order.

## Evaluating the demogrant proposal

Evaluation of Buchanan’s demogrant proposal faces the significant obstacle that the proposal has never been implemented. Clearly, such a radical reform to both the tax and the benefit side of the social security system pushes at the boundaries of what is politically feasible in contemporary democracies. Implementation of the demogrant would surely require assimilation of frequently distinct political concerns regarding the justice and efficacy of tax and welfare policy. Hence, all UBI schemes face practical bottlenecks of implementation that jeopardise their political feasibility (Boettke and Martin 2012; De Wispelaere and Stirton 2012). Nevertheless, some important evaluative points can be made.

Like all proposals for a universal basic income, the demogrant would seem to open the possibility of a significant rise in government expenditure. Brennan and Buchanan (1980: 189–190) noted that the application of the generality principle to taxation could increase the overall tax burden because, absent the disproportionate imposition of costs on wealthy minorities, the point at which the marginal benefits of taxation exceeded the marginal costs for the median voter could be at a rate that increased the overall government budget.

Indeed, Berggren (2000) warned that the demogrant proposal could lead to a ‘fiscal explosion’ as the median voter logically has an incentive to maximise the size of the demogrant essentially without limit, at least assuming that the required imposition of prohibitive taxation would not (be perceived to) reduce productivity and therefore impose indirect costs on demogrant recipients. Accordingly, Berggren argued that the generality principle should be augmented with a constitutional rule stating that public expenditures as a share of GDP must not rise. Berggren argued that once the fear of significant budgetary expansion had been removed generality should become more attractive than non-generality for most citizens.

However, while some means of assuring that the application of generality does not lead to a fiscal explosion must be important, if a demogrant led to a significant reassignment of the tax burden then this is also likely to prove unpopular among citizens. A basic principle of public finance is that significant reassignments of the tax burden tend to be unpopular with those asked to pay more and for this reason governments prefer to raise additional revenue through incremental variation of existing tax rates rather than via the introduction of new taxes (or the abolition of existing ones) (Gibson and Watt 1994). The example of the UK Poll Tax, which was introduced in 1989 and 1990 with the intention of introducing some elements of generality into the financing of UK local government, may be instructive. It is estimated that more than 60 per cent of UK households experienced a change in their contribution to local taxation of more than 20 per cent—some gaining, some losing—as a result of the new tax. A vociferous political campaign was launched against the tax, including widespread non-payment, eventually leading to the downfall of Prime Minister Margaret Thatcher and the abolition of the Poll Tax. Consequently, this attempt to introduce some elements of generality into the UK tax system has been widely considered an unmitigated failure (Gibson and Watt 1994; Meadowcroft 2006).

Buchanan’s argument for the demogrant rests on the ethical desirability of the application of the generality principle and also on the belief that the cessation of rent seeking would lead to significant welfare gains that in the long-run would make everyone better-off. The rates of flat tax and demogrant would determine the precise distribution of benefits and burdens and it must be possible that there could be significant losers (at least in the short-term) if a demogrant was introduced in a contemporary democracy. There is a risk that a demogrant could be an economic shock that created social and political unrest that replicated some of the actions associated with rent seeking that the demogrant is intended to end. At the same time, a ‘watered down’ demogrant might be more palatable to voters and easier to implement but it might lose many (if not most) of the advantages of the more stringent model. This is arguably what happened to the Negative Income Tax as shown by the fact that even Milton Friedman turned against Nixon’s FAP bill after it became clear that ‘the negative income tax would be layered on top of other programs’ and be encumbered with conditionalities (Moffitt 2003: 122).

Finally, the demogrant proposal rests upon the ethics of the generality principle and the efficiency gains associated with the end of rent seeking, but it might be argued that, in common with all guaranteed income policies, it raises some ethically-troubling incentive and moral hazard problems. A guaranteed basic income without any qualifying obligation enables some people to live at the expense of others, including able-bodied people who refuse to work. One of the most important advocates of a universal basic income, Philippe Van Parijs (1991; 1995, Chapters 4 and 5), has argued that lazy individuals who wanted to do nothing more than surf every day should be entitled to a guaranteed basic income even though they made no productive contribution to the wealth creation that funded their lifestyles because a goal of a basic income was to free people from the ‘coercion’ they experienced in capitalist labour markets. Some republicans have similarly argued for UBI as a tool against economic ‘domination’ (Pettit 2007; Zwolinski 2019). Hence, while a demogrant may end the complex rent seeking processes associated with the welfare state, it would seem to create new rents that people could obtain simply by choosing to forego work and instead spend the day surfing at the beach while others laboured to fund their lifestyles. Whether this exploitation objection is persuasive depends on the weight of the ethical and economic arguments that seek to overcome it (White 1997, 2006). One controversial but plausible way to counter this objection would be to accept the ethical claim that people have a right to a guaranteed minimum endowment of resources—a view that could be consistent with Buchanan’s (1975) conception of political rights—in which case the recipients of a demogrant cannot be said to exploit anyone when they enjoy their basic income (See e.g. Fleischer and Lehto 2019; Steiner 2016; Widerquist 2013).

Buchanan and Congleton (1998: 40–41) set out another plausible but quite different counterargument that accepts the charge that the demogrant system encourages exploitation and unfairness but argues that the amount of exploitation and unfairness under a non-universal, non-general benefit system is, in fact, comparatively *greater*. Buchanan and Congleton’s comparative institutional judgement was that while incentives for rent seeking must exist in any transfer system, the incentives for rent seeking under conditions of generality were minimal by comparison with the incentives that emerged under nonconstrained majority voting. Buchanan and Congleton (1998: 40–41) argued that in the context of the demogrant individuals ‘retain some incentives to invest in efforts to convince others to support their own preferred positions, but there remain no incentives for persons to seek membership in majority coalitions that are aimed specifically at discriminatory or differential advantage to the majority at the expense of members of the minority’. In other words, while under the demogrant an individual may choose to exit from the labour market and live relatively frugally at the expense of others, it would no longer be possible for individuals to create majority coalitions to use the tax and benefit system to extract significant resources from a minority of the population. Accordingly, while the demogrant would not remove all exploitation via the political process, it would significantly reduce the possible scope and scale of political exploitation.

## Conclusion

James M. Buchanan’s proposal for a demogrant has been unduly neglected by scholars working in public choice and constitutional political economy. The demogrant is a logical application of Buchanan’s theoretical framework of politics as exchange in the absence of reform to the basic institutions of majoritarian democracy. The demogrant offers a means of ending the majority cycle and the rent seeking generated by targeted and means-tested benefit systems. As such, it offers a model of welfare without rent seeking—a constitutional welfare state.

The application of the principles of generality and non discrimination to the tax *and* transfer sides of the system, together with the constitutionalization of those principles, would seem to avoid many of the potential problems of spiralling costs and exploitation of the productive by the lazy often ascribed to basic income policies, at least when combined with something like Berggren’s (2000) proposal for a linked constitutional rule to prevent the expansion of the government budget. Nonetheless, it may be hard to bring together an effective political coalition to implement a lasting constitutional demogrant. In addition, we believe that the transaction costs associated with a significant reassignment of the tax burden (and a significant change to the institutional structure of the welfare state) undoubtedly require further analysis before any attempt was made to implement the demogrant.

A plausible path forward is to encourage experimentation and reform in welfare policy building on the strengths and weaknesses of past and current UBI experiments. On a theoretical level, the ethical, economic, and political dimensions of the demogrant merit more research. A fuller theoretical assessment of *tax*-*and*-*transfer symmetry* and *constitutionalization*—the two unique features of the demogrant model—could illuminate the unique merits and demerits of the demogrant over UBI and NIT. So, rescuing the demogrant model from relative obscurity will be a fruitful future task of applied constitutional political economy and public choice.

## Footnotes

1 https://data.oecd.org/socialexp/social-spending.htm.

2 Buchanan (1992) rejected the idea that academics should hand-down policy prescriptions to the public or policy-makers (Meadowcroft and Ruger 2014). The principal exception was the idea of a balanced budget, which Buchanan explicitly advocated, almost certainly on the basis that public debt necessarily imposed costs on people unable to consent to its creation and therefore it was always illegitimate.

3 An English language summary of the final government report can be found here: https://www.kela.fi/web/en/news-archive/-/asset_publisher/lN08GY2nIrZo/content/results-of-the-basic-income-experiment-small-employment-effects-better-perceived-economic-security-and-mental-wellbeing.
